# Supplementary material for: Complexome profiling of the Chlamydomonas psb28 mutant reveals TEF5 as an early PSII assembly factor
Source: Plant Cell. 2025 Mar 17;37(6):koaf055. doi: 10.1093/plcell/koaf055 (PMC12164590; doi:10.1093/plcell/koaf055)
Supplement: koaf055_Supplementary_Data [file koaf055_supplementary_data.zip › psb28_interactive.zip!/plots/intensity/qualityPlots/peptides/_Cre13.g581850.t1.2.html]

### Cre13.g581850.t1.2

Trivial name:   
SeqCoverage [median]: 22.40  
Mass: 72.42 kDa  
  
 

| Sequence | MissedCleavage | Mass [Da] | wtIntensity | muIntensity | Score |
| --- | --- | --- | --- | --- | --- |
| AATALAPSTR | 0 | 957.52 | 1488.28 | 4565.87 | 133.57 |
| AGQVLANRPDIVR | 1 | 1407.79 | 0.00 | 607.89 | 69.98 |
| ELLTVLGPSFIK | 0 | 1315.78 | 399.66 | 0.00 | 80.69 |
| GPGDAALVALR | 0 | 1038.58 | 0.00 | 765.81 | 76.83 |
| GQQLSDFNFR | 0 | 1210.57 | 0.00 | 2054.05 | 112.84 |
| LGFLAPGTDITPIVPALEK | 0 | 1951.10 | 347.60 | 0.00 | 82.07 |
| LLEELDYTQEAK | 0 | 1450.72 | 0.00 | 2503.79 | 91.86 |
| LVQNDINPQK | 0 | 1167.62 | 662.88 | 2762.33 | 87.31 |
| NFRDDPTVK | 1 | 1090.54 | 0.00 | 262.01 | 37.56 |
| RLEEVFSSISERPVAAASLGQVYK | 2 | 2635.40 | 0.00 | 1096.60 | 24.28 |
| VLRGPGDAALVALR | 1 | 1406.84 | 0.00 | 1923.64 | 52.58 |
| VQRPGVEPTILR | 1 | 1363.79 | 467.08 | 3234.15 | 60.26 |
|  |  |  | 3365.5 | 19776.1 |  |
